# A Case of Neonatal Urosepsis with Multifocal Osteoarthritis: Could Ultrasonography Change the Clinical Course?

**DOI:** 10.5812/iranjradiol.4079

**Published:** 2013-08-30

**Authors:** Giovanni Ottonello, Angelica Dessì, Maria Elisabetta Trudu, Carmela Porcu, Vassilios Fanos

**Affiliations:** 1 Neonatal Intensive Care Unit, Puericulture Institute and Neonatal Section, University of Cagliari, Italy; 2 Institute of Radiology, Azienda Ospedaliero Universitaria di Cagliari, Italy

**Keywords:** Infant, Newborn, Osteomyelitis, Pyelonephritis, Pyelectasis, Ultrasonography

## Abstract

An eleven-day boy neonate with a fetal anamnesis of grade 1 bilateral hydronephrosis according to the grading of the Society for Fetal Urology (SFU), came to our attention for an acute osteoarthritis secondary to urosepsis. In the urological follow-up, a severe bilateral vesico-ureteral reflux (VUR) was diagnosed. An early post-natal, reno-vesicle ultrasound evaluation could have changed the clinical course of our patient.

## 1. Introduction

It is known that bacteremia is a relatively frequent complication of acute pyelonephritis in the neonate and the unweaned infant, while in the literature cases of hematic diffusion and suppurative complications such as osteoarthritis (1-3) are quite rare. To date, only two cases of neonatal osteoarthritis have been reported in which gram-negative germs were isolated in the synovial liquid and the association with a vesico-ureteral reflux (VUR) (1, 2) was documented. We describe a clinical case of osteoarthritis and urosepsis that recently came to our attention. Certain considerations from the epicrisis of the current case emphasize the importance of the timing of the first post-natal ultrasonography in newborns with mild-to-moderate fetal pyelectasis (5-10 mm; 10-15 mm).

## 2. Case Presentation

**Figure 1.**
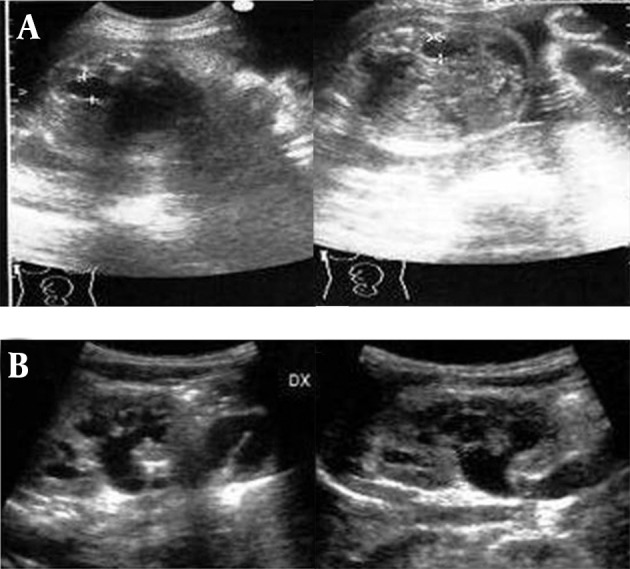
A. Fetal ultrasound at 31 weeks of gestational age demonstrating bilateral hydronephrosis grade 1 (SFU). B, Ultrasonography of the right and left kidney on admission showing bilateral ureterohydronephrosis grade 3 (SFU)

An eleven-day boy neonate was hospitalized due to the onset of acute hyperemia and swelling of the right medial malleolus, swelling of the right knee and immobility of the leg. The fetal anamnesis at the 21st week and 31st week of gestational life revealed a grade 1 bilateral hydronephrosis (SFU) (Figure 1 A), with no other pathology reported. While hospitalized, no renovesical ultrasonography was performed, but a post-release checkup was advised after the first week and in any case within the first month of life. 

**Figure 2.**
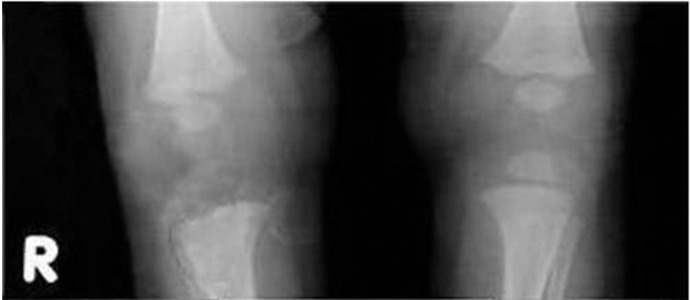
X-ray of the lower limbs 15 days after admission (1 month of age) shows osteolysis of the upper metaphysis of the right tibia.

**Figure 3.**
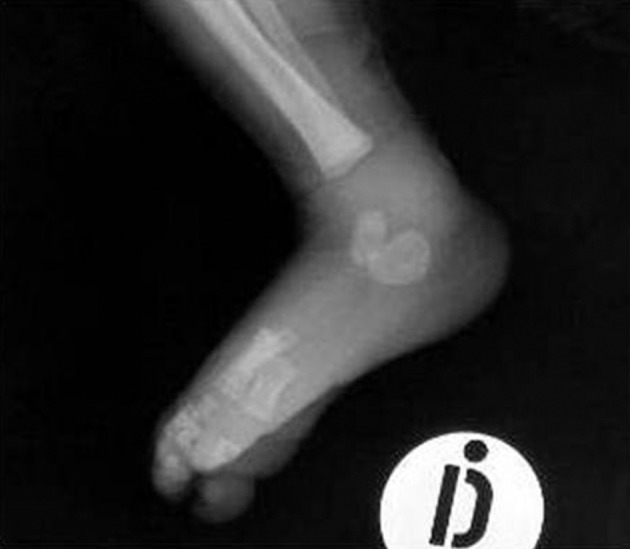
X-ray of the right ankle on admission at 11 days of age

**Figure 4.**
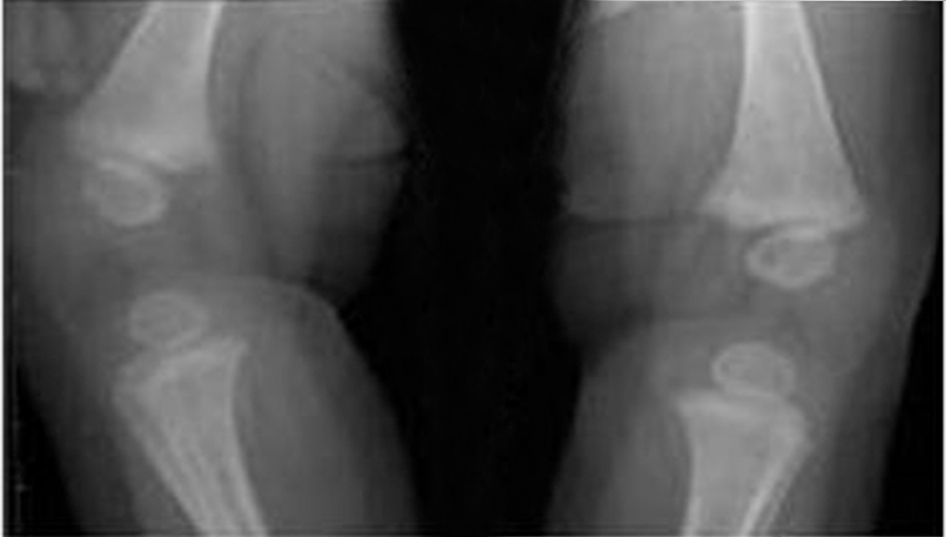
X-ray control at 4 months of age: resolution area of osteolysis

On admission, the laboratory analyses performed urgently for suspected osteoarthritis were indicative of the presence of a bacterial infection; 25,500/mm3, white cells with neutrophilia; C-reactive protein, 15 mg/dl; Procalcitonin, 21.7 ng/ml. Moreover, the urine unexpectedly appeared quite turbid, with nitrites and leukocyte esterase positive in deep stick analysis, pyuria and hematuria in the sediment. While urine, blood and synovial liquid cultures were performed, preparations were made for a renovesicle ultrasonography. It revealed a grade 3 bilateral hydronephrosis (SFU) with dilated and twisted ureters (Figure 1 B). Fifteen days after admission, radiography of the right limb showed a zone of osteolysis at the level of the upper tibial metaphysis (Figure 2) and a normal ankle (Figure 3). An empirical intravenous therapy was started with a double antibiotic (ampicillin-sulbactam and gentamicine) and on the following days the blood, urine and synovial liquid cultures were found positive for Escherichia coli with the same antibiotic sensitivity. According to the antibiogram, the empirical therapy was changed to targeted antibiotics and protracted for an overall period of 32 days. Clinically, there was a gradual improvement in the overall clinical picture and the functional recovery of the femorotibial articulation (Figure 4). 

**Figure 5.**
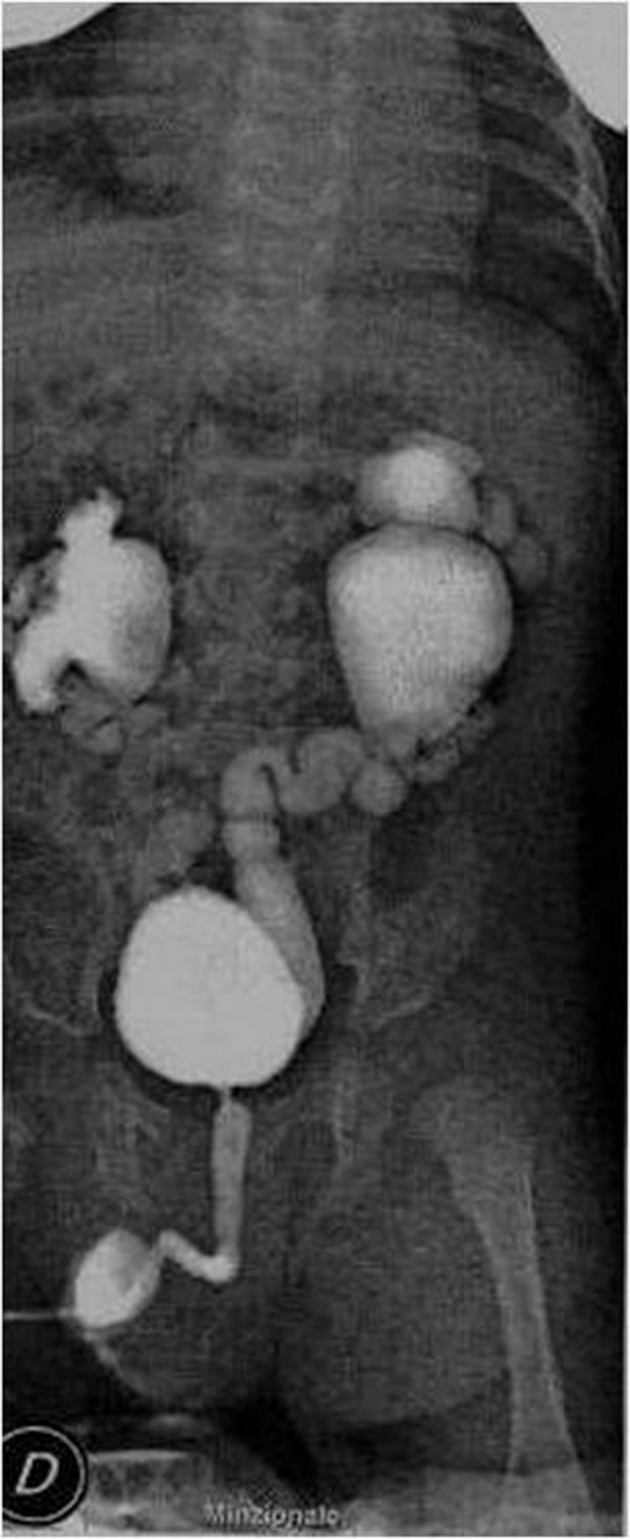
Bilateral VUR with urine cystoureterography (2 months of age)

In the follow-up, a voiding cystourethrography revealed a passive vesico-ureteral reflux (VUR) grade 4 on the right side and grade 5 on the left side (Figure 5). 

## 3. Discussion

To date, only two cases of osteoarthritis and neonatal urinary infection in patients with VUR have been reported in international literature (1, 2). In the present case, the bacteria identified in the cultures were gram negative that are rarely responsible for neonatal osteoarthritis, which is usually caused by gram positive bacteria. Involvement of the joints appeared secondary to an early gram negative urinary tract infection (the most common germs found in acute pyelonephritis) complicated by septic diffusion and subsequent localization in the joint. From the epicrisis of the current clinical case, the important recommended point is the late performance of the first post-natal urinary tract ultrasound examination for the patient instead of an early examination because the newborn will have proper diuresis and rehydration that lowers the probability of false negative results (4) was the cause of different indications as concerns the time of the examination: third day of life, but also the fifth, or after the first week. It is to be kept in mind that the first postnatal verification is useful not only in confirming the pathology found during pregnancy, but also in evaluating changes with time or identifying aspects that were not revealed in the fetal period. Several studies have reported that ultrasound sensitivity is superior in the neonatal period compared to the prenatal period (5).

The clinical case presented herein shows once again that a moderate bilateral pyelectasis of the fetus may conceal the presence of a severe VUR (as has often been demonstrated for mild cases of pyelectasis). It is particularly important to establish the proper period for the first standardized postnatal sonographic screening to prevent delay in beginning antibiotic prophylaxis if necessary. In our clinical case, an early ultrasonography would have suggested an antibiotic prophylaxis that in the case of VUR represents a therapy in the neonatal period that could maintain a better clinical course for the patient.
